# Integrating Radiomics and Artificial Intelligence (AI) in Stereotactic Body Radiotherapy (SBRT)/Stereotactic Radiosurgery (SRS): Predictive Tools for Tailored Cancer Care

**DOI:** 10.3390/cancers17172906

**Published:** 2025-09-04

**Authors:** Ilaria Morelli, Marco Banini, Daniela Greto, Luca Visani, Pietro Garlatti, Mauro Loi, Michele Aquilano, Marianna Valzano, Viola Salvestrini, Niccolò Bertini, Andrea Lastrucci, Stefano Tamberi, Lorenzo Livi, Isacco Desideri

**Affiliations:** 1 Oncology Unit, Santa Maria delle Croci Hospital, AUSL della Romagna, 48121 Ravenna, Italy; ilaria.morelli2@auslromagna.it (I.M.);; 2 Radiation Oncology Unit, Careggi University Hospital, 50134 Florence, Italyaquilanomichele@gmail.com (M.A.);; 3 CyberKnife Center, Istituto Fiorentino di Cura ed Assistenza, 50139 Florence, Italy; 4 Department of Allied Health Professions, Azienda Ospedaliero-Universitaria Careggi, 50134 Florence, Italy; 5 Department of Biomedical, Experimental and Clinical Sciences “Mario Serio”, University of Florence, 50121 Florence, Italy

**Keywords:** stereotactic body radiotherapy, stereotactic radiosurgery, artificial intelligence, outcomes prediction, treatment toxicity

## Abstract

**Purpose:** This systematic review aims to analyze the literature on the application of AI in predicting patient outcomes and treatment-related toxicity in those undergoing SBRT or SRS across heterogeneous tumor sites. **Materials and methods:** Our review conformed to the Preferred Reporting Items for Systematic Reviews and Meta-Analyses. PubMed, EMBASE and Scopus were systematically searched for English-language human studies evaluating AI for outcome and toxicity prediction in patients undergoing SBRT or SRS for solid tumors. Search terms included (“Stereotactic Body Radiotherapy” OR “SBRT” OR “Stereotactic Radiosurgery” OR “SRS” OR “Stereotactic Ablative Radiotherapy” OR “SABR”) AND (“Artificial Intelligence” OR “AI” OR “Machine Learning” OR “Deep Learning” OR “Radiomics”) AND (“Response Prediction” OR “Response to Treatment” OR “Outcome Prediction”) AND (“Toxicity” OR “Side Effects” OR “Treatment Toxicities” OR “Adverse Events”). **Results:** The search yielded 29 eligible retrospective studies, published between 2020 and 2025. Eight studies addressed early-stage primary lung cancer, highlighting the potential of AI-based models in predicting radiation-induced pneumonitis, fibrosis and local control. Five studies investigated AI models for predicting hepatobiliary toxicity following SBRT for liver tumors. Sixteen studies involved SRS-treated patients with brain metastases or benign intracranial neoplasms (e.g., arteriovenous malformations, vestibular schwannomas, meningiomas), exploring AI algorithms for predicting treatment response and radiation-induced changes. In the results, AI might have been exploited to both reaffirm already known clinical predictors and to identify novel imaging, dosimetric or biological biomarkers. Examples include predicting radiation pneumonitis in lung cancer, residual liver function in hepatic tumors and local recurrence in brain metastases, thus supporting tailored treatment decisions. **Conclusions:** Combining AI with SBRT could greatly enhance personalized cancer care by predicting patient-specific outcomes and toxicity. AI models analyze complex datasets, including imaging and clinical data, to identify patterns that traditional methods may miss, thus enabling more accurate risk stratification and reducing variability in treatment planning. With further research and clinical validation, this integration could make radiotherapy safer, more effective and contribute to advancement in precision oncology.

## Simple Summary

Stereotactic body radiotherapy (SBRT) and stereotactic radiosurgery (SRS) are advanced treatments used to precisely target tumors in different parts of the body. However, predicting how well patients will respond and who may experience side effects remains challenging. This study review explores how Artificial Intelligence (AI) can help improve these predictions. By analyzing medical images and patient data, AI tools may identify patterns that clinicians might not notice, helping to tailor treatment and reduce harm. Our findings suggest that using AI alongside SBRT and SRS could lead to safer and more effective cancer care.

## 1. Introduction

### 1.1. Stereotactic Body Radiotherapy (SBRT): An Overview

SBRT is defined as a method of external beam radiotherapy (EBRT) that precisely delivers a high dose of radiation in one or few treatment fractions to an extracranial target [1,2]. In SBRT workflow, radiation doses are at least equivalent to radical doses in conventional fractionation and are delivered in few fractions (usually in a maximum of ten). Since SBRT requires an accurately targeted tumor area in the body, the target is usually localized using tumor site specific imaging modalities and it is separately delineated from critical organs at risk (OARs), in absence of diffuse infiltration. Only the macroscopic target and small, immediately adjacent, volumes of potential microscopic spread are treated with SBRT.

SBRT, which can be effectively applied using both photons and particle therapy, can be successfully carried out either with traditional linear accelerators equipped with appropriate image-guidance technology, with accelerators specifically adapted for SBRT or with dedicated delivery systems. In this regard, the emerging role of Magnetic Resonance Image (MRI)-Linac is becoming increasingly important in the treatment of both primary tumors and oligometastases. The main advantages of MRI-Linac are superior soft-tissue visualization, real-time motion monitoring and the ability to adapt treatment online, which together help to reduce the irradiation of surrounding organs at risk and limit radiation-induced side effects. The potential for dose escalation is site-specific: for some tumors located adjacent to critical structures (e.g., pancreas or liver), MRI-Linac may create opportunities to safely deliver higher doses that would not be feasible with conventional linacs. In contrast, for more common targets such as lung or prostate, the benefits of MRI-Linac are less related to dose escalation and more to accurate target localization and intra-fraction monitoring, ensuring standard therapeutic doses can be delivered with greater precision and confidence. [3,4].

SBRT is often used to treat tumors from lungs [5], liver [6], spine [7], prostate [8] and pancreas [9], and it represents a valid alternative for those who may not be good candidates for surgery due to tumor location or other health concerns. In the setting of oligometastatic disease [10], SBRT represents a cornerstone for the treatment of both primary and metastatic sites [4,11,12,13], either alone or in combination with systemic therapy.

When discussing intracranial targets, we usually refer to Stereotactic Radiosurgery (SRS) [14]. While SBRT and SRS are similar in their use of highly precise, high-dose radiation over one/a few treatment sessions, the distinction lies in their target areas, with SRS commonly used for the management of brain metastases [15,16], arteriovenous malformations (AVMs) and some benign tumors (e.g., acoustic neuromas).

### 1.2. Artificial Intelligence (AI): Definition and Application in Radiation Oncology Practice

There is no universally agreed-upon definition of AI; however, the High-Level Expert Group on Artificial Intelligence (AI HLEG) of the European Commission (EC) defines AI as “*systems that exhibit intelligent behavior by interpreting their environment and making decisions—at least partially autonomously—to accomplish specific objectives.*” [17].

AI is a broad concept that includes a variety of technologies designed to enable machines to perform tasks that typically require human intelligence, such as problem-solving, decision-making and speech or image recognition. For this purpose, advanced machine learning (ML) algorithms can simulate the human ability to learn [18].

In the Radiation Oncology setting, AI has been recently playing a crucial role across many aspects of cancer care, from diagnosis [19,20,21,22,23] and treatment planning [19,24,25,26] to patient stratification [27,28] and monitoring [29] and outcome prediction [23,30]. Specifically, AI can assist in the delineation of tumors and OARs by automating the segmentation process and can optimize dose distribution, thus ensuring tumors receive adequate treatment while minimizing damage to surrounding healthy tissues. AI is also being actively investigated for real-time monitoring and adaptive radiation delivery based on tumor changes or patient movement; however, these applications are not yet implemented in routine clinical practice and remain an area of ongoing research [31,32]. In addition, AI can be integrated into clinical workflows for patient stratification, development of personalized treatment plans and outcome and toxicity prediction. When dealing with Radiomics, we refer to a non-invasive and emerging technique that in an initial phase involves the extraction of quantitative features from routine medical imaging (e.g., MRI or computed tomography (CT) scans), followed by feature selection and analysis in relation to clinical data [33,34]. These features typically capture tumor characteristics such as signal intensity, shape and texture patterns, thereby providing reader-independent data for predictive modeling [35]. It must be specified that the quality of radiomic features is strongly influenced by image sharpness and that motion blurring (e.g., due to respiration) can significantly degrade their robustness and reproducibility. Therefore, radiomic analyses are typically performed on images acquired at a single phase of the breathing cycle (e.g., 3D-CT or a selected phase of 4D-CT), rather than on average intensity projections or CBCT scans acquired in free breathing [36,37,38].

More in details, radiomic features can be categorized into different groups based on their mathematical and statistical properties [39]:

- Shape Features: These features describe the geometric properties of the Regions of Interest (ROIs) or Volumes of Interest (VOIs), including volume, diameter, sphericity and compactness.
- First-Order (Histogram) Features: These features characterize the distribution of voxel intensities within the segmented region, encompassing statistical measures such as mean, median, skewness and kurtosis.
- Second-Order Texture Features [40,41]: These features capture the statistical relationships between neighboring voxels or voxel groups within the lesion. For this purpose, various matrices can be employed for feature extraction, including
- Gray-Level Co-Occurrence Matrix (GLCM): It analyzes pixel pair distribution within the image.
- Gray-Level Run-Length Matrix (GLRLM): It quantifies the length of consecutive voxels with identical intensity along a specific direction.
- Neighborhood Gray-Level Difference Matrix (NGLDM): It measures the difference between a voxel’s intensity and the average intensity of its neighboring voxels within a defined distance.
- Higher-Order Texture Features: Derived through additional mathematical transformations that emphasize specific aspects of the ROI, enabling extraction of a broader range of features.

AI is emerging as a cornerstone of precision medicine, particularly through its applications in Radiomics. When integrated with deep learning (DL) approaches, which can identify patterns in high-dimensional and noisy data, making it well-suited for analyzing -*omics* datasets [42], Radiomics indeed can manage a huge number of data and directly analyze the imaging to create the appropriate features for cancer diagnosis and molecular profile [43,44,45], outcomes prediction [46,47,48,49] and response to treatment [50,51,52].

Given the growing integration of AI into clinical radiotherapy workflows, particularly in SBRT and SRS, many studies have explored its utility in areas such as target and OARs segmentation, treatment planning and treatment delivery [53]. Specifically, for what concerns treatment planning, Kumar et al. [54] reported the results on dosimetric optimizations in VMAT using the Monaco system, highlighting the importance of precise treatment planning, which can be further enhanced by AI-driven radiomics in SBRT/SRS.

However, there is an increasing interest in the potential of AI to support also prediction of patient outcomes and treatment-related toxicities, which remain a critical aspect of personalized care. The aim of this systematic review is therefore to comprehensively map and synthesize the existing literature on the application of AI—particularly Radiomics and DL approaches—for outcome prediction and toxicity assessment in patients undergoing SBRT and SRS across different tumor sites. By providing an updated overview of current evidence and research gaps, this review aims to clarify the evolving role of AI in enhancing precision medicine, decision-making and quality of care in stereotactic radiotherapy.

## 2. Materials and Methods

This systematic review conformed to the Preferred Reporting Items for Systematic Reviews and Meta-Analyses Reviews [55]. The PubMed, EMBASE and Scopus databases were comprehensively searched to identify published studies assessing the role of AI in outcome prediction and toxicity assessment in patients undergoing SBRT or SRS for mixed-sites solid tumors. Search terms included (“Stereotactic Body Radiotherapy” OR “SBRT” OR “Stereotactic Radiosurgery” OR “SRS” OR “Stereotactic Ablative Radiotherapy” OR “SABR”) AND (“Artificial Intelligence” OR “AI” OR “Machine Learning” OR “Deep Learning” OR “Radiomics”) AND (“Response Prediction” OR “Response to Treatment” OR “Outcome Prediction”) AND (“Toxicity” OR “Side Effects” OR “Treatment Toxicities” OR “Adverse Effects”).

After completing the literature search, all records were uploaded onto a dedicated reference management software. Duplicates were first identified and removed using the software’s automatic detection tool and then verified through additional manual checking. Subsequently, two reviewers (I.M., I.D.) independently screened the titles and abstracts of the remaining studies to exclude non-relevant publications. Then, the full texts of eligible articles were obtained and evaluated for potential inclusion by the same reviewers. Discrepancies between reviewers were solved in a case-by-case discussion among co-authors. The same two reviewers independently performed data extraction and entered the information into a custom-designed electronic database created for this review. This database facilitated efficient tracking, updates and data export for analysis. The entire research team reviewed the final extracted data to identify discrepancies and ensure accuracy. Following the data extraction process, the team synthesized and discussed the findings. A standardized data extraction form was used to verify the included studies and capture key information, such as first author, year of publication, treatment site, sample size, outcome assessed, predictive performance of the developed model and main findings.

Selected papers were in English and included only publications in human subjects. Other inclusion criteria involved (a) tumors treated by SBRT or SRS; (b) outcomes and/or treatment-related toxicities prediction as primary endpoint; (c) employment of AI-based models for outcome/toxicity prediction; and (d) retrievable English full text. Studies meeting any one of the following criteria were excluded: (a) studies involving radiation techniques different from SBRT or SRS; (b) studies focusing on different outcomes than treatment response and/or side effects prediction (e.g., treatment planning or treatment delivery); and (c) studies published as abstracts, case series, letters, reviews or supplements.

The quality of each study was assessed by the same authors based on the Newcastle-Ottawa Scale (NOS) criteria, which aims at evaluating the quality of nonrandomized trials. The quality of the studies is based on three domains: the selection (maximum score 4), the comparability (maximum score 2) and the exposure (maximum score 3) assessment. The total score can reach up to a maximum of 9 and a score ≥7 is considered an indicator of high quality. In case of any discrepancies in the quality assessment process, another investigator had been consulted.

**Figure 1:**
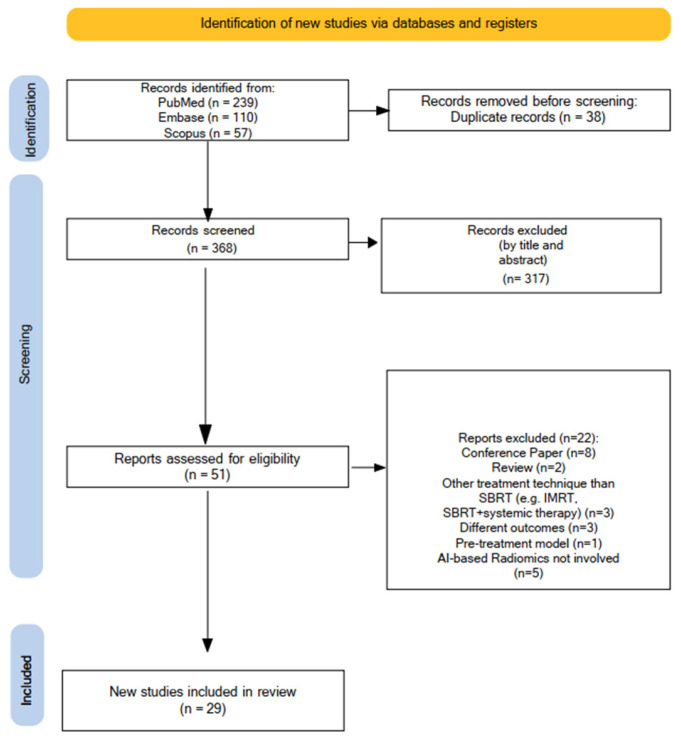
PRISMA flow diagram illustrating the subsequent phases of the review search and the study selection process.

PRISMA flowchart illustrating the various phases of the review search and the study selection process is reported in Figure 1. A completed PRISMA checklist is provided as a Supplementary File.

This study was registered on PROSPERO (Registration No. ID 1112365).

## 3. Results

Our initial search retrieved 406 published papers between 1985 and 2025 (PubMed: n = 239; Scopus: n = 57; Embase: n = 110); after duplicate removal (n = 38), 368 works were first screened by title and abstract. Among 51 papers assessed for eligibility, 22 were further excluded (conference paper n = 8; reviews n = 2; other treatment technique than SBRT n = 3; different outcomes n = 3; pre-treatment model n = 1; AI-based radiomics not involved n = 5).

In the end, 29 works were included in the present review, and their main characteristics are reported in Table 1, Table 2 and Table 3.

Most predictive models were radiomic-based, with features extracted from CT or MRI scans. Selected papers were divided according to treatment site (lung, hepatobiliary, CNS). For each paper, first author, publication year, imaging modality, tumor histology, number of enrolled patients, assessed outcomes, model predictive performance and main findings were reported. All included studies were retrospective. In most studies, model performance was evaluated using Receiver Operating Characteristic (ROC) curves and the corresponding Area Under the Curve (AUC) values (ranging from 0 to 1), where higher AUC scores reflect greater predictive accuracy. Also, the F1 score was used to evaluate model accuracy, particularly when dealing with imbalanced datasets. The F1 score ranges from 0 to 1, with 1 being the best possible score, indicating perfect precision and recall, and 0 being the worst.

The quality of studies assessed using NOS is shown in Table 4. The NOS scores ranged from 5 to 9, with higher scores indicating better methodological quality and lower risk of bias. Out of the total studies, twenty-one (approximately 72%) scored between 7 and 9, indicating a low risk of bias and generally high methodological quality. On the other hand, eight studies (approximately 28%) scored between 5 and 6, reflecting a moderate risk of bias. No studies scored below 5, suggesting that none were classified as having a high risk of bias. Overall, the majority of studies included in the analysis demonstrated low risk of bias, supporting the reliability of the findings. However, a notable proportion of studies presented moderate risk, which should be considered when interpreting the results.

### 3.1. Integration Between AI and SBRT for Treatment Assessment and Outcomes Prediction in Lung Cancer

**Figure 2:**
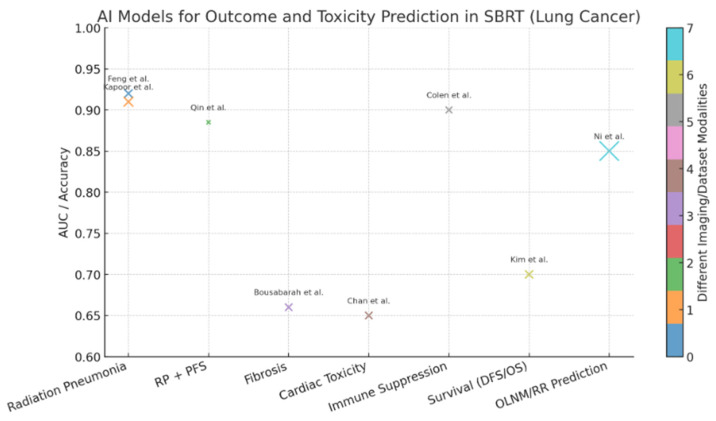
Bubble chart summarizing the main findings of studies evaluating AI models in lung cancer patients treated with SBRT [56,57,58,59,60,61,62,63]. The *x-axis* represents the predicted clinical endpoints (treatment-related toxicities: radiation pneumonitis, fibrosis, immunosuppression; and oncologic outcomes: OS, PFS, local control). The *y-axis* indicates the predictive performance reported (AUC or accuracy). Bubble size is proportional to the number of patients included in each study, and bubble colors distinguish the different input modalities used (e.g., CT-based radiomics, CT + DVH features, CBCT radiomics or hybrid dosimetric–radiomic models). Labels correspond to the first author of each study. This visualization provides an at-a-glance overview of the heterogeneity of AI approaches, their sample sizes and performance across toxicity and outcome prediction tasks.

Eight selected papers from 2020 and 2024 evaluated the application of AI models for outcome and toxicity prediction in lung cancer patients undergoing SBRT. Specifically, four studies focused on the development of radiation-induced toxicity (pneumonia or fibrosis), whereas five works investigated potential correlation with overall survival (OS), progression-free survival (PFS) and local control. One single study focused on the development of immunity suppression following SBRT. CT-based radiomic models were the most exploited (Table 1, Figure 2).

**Table 1:** Artificial Intelligence-based models for outcomes assessment and toxicity prediction in patients undergoing SBRT for lung cancer.

First Author	Year	Imaging Modality (If Radiomic Model)	Type of Used AI	N. Patients	Outcomes Assessed	Predictive Performance (AUC, Accuracy, Sensitivity)	Main Findings
Feng A. [56]	2024	CT, Dosiomics	Deep Learning	140	RP	CT-based model AUC: 0.791 CT + DVH model AUC: 0.809 CT + Rtdose model AUC: 0.907 Hybrid (CT+ DVH + Rtdose) model AUC: 0.920	Dosiomic features can improve the performance of the predictive model for symptomatic RP compared with that obtained with the CT + DVH model
Kapoor R. [57]	2023	CT	Deep Learning (CNN)	193	RP	3D-DenseNet model: F1score 0.81, AUC 0.91 for 3-class prediction (No RP, RP1, RP 2); F1 score 0.77, AUC 0.84 for 2-class prediction (no RP, yes RP)	3D CNN models (especially DenseNet-121) effectively predict the risk of RP using CT scans and radiation dose data
Qin Q. [58]	2020	CT	Machine Learning	34	PFS, lung toxicity	Planning CT radiomic features-based model: AUC 0.913 (PFS) and 0.832 (toxicity). Radiomic features of the CBCTs plus planning CT (planning CT + CBCT1 + CBCTmid + CBCTlast) model: AUC 0.885 (PFS) and 0.885 (toxicity)	Both pretreatment CT and CBCT radiomic features could predict disease progression and lung injury. A model with CBCT plus pretreatment CT radiomic features might improve the prediction of lung toxicity in comparison with a model with pretreatment CT features alone
Bousabarah K. [59]	2021	CT	Machine Learning	110 (training cohort); 71 (test cohort)	LC, OS, DFS, lung fibrosis	Radiomic models CI (training cohort) for OS, DFS and LC: 0.77–0.99, *p* < 0.005 Radiomic models CI (test cohort) for OS, DFS and LC: 0.36–0.49 Combined models CI for lung fibrosis (training set): 0.71–0.79, *p* < 0.005 and in the test set CI for lung fibrosis (test cohort): 0.59–0.66, *p* < 0.05	The best-performing model included GTV-Dmean, PTV-D95%, Lung-D1ml, age and 7 radiomic features (CI 0.66, *p* < 0.03). Radiomics analysis can be used for prediction of local lung injury after SBRT of NSCLC
Chan ST. [60]	2020	Cardiac dosimetry data	ANN	112	OS	ANN test accuracy: 64.7%	Cardiac substructure dosimetry, esp. RV V10Gy, is associated with OS; ANN model predicted survival
Colen J. [61]	2024	DICOM-based blood-dose simulation	Simulation-driven, mechanistic AI	64	RIIS, ALC	An algorithm using DICOM data from RT treatment plans, dose maps, patient CT scans and organ delineations predicted the fraction of lymphocytes killed during SBRT treatment with 81% sensitivity and 98% specificity	The algorithm effectively predicted RIIS in patients undergoing SBRT for lung cancer, with strong accuracy and the potential for real-time integration into treatment planning
Kim H. [62]	2021	CT	Deep Learning	135	LRFS, DFS, OS	Deep learning model AUC for LRFS, DFS and OS was 0.72, 0.70 and 0.66, respectively. The model provided useful independent information beyond clinical factors	A CT-based deep learning model, even though designed for surgical patients, effective at predicting outcomes for patients undergoing SBRT
Ni J. [63]	2024	CT	Machine Learning	769 (training + validation); 213 (SBRT cohort)	Occult LN metastasis; Regional recurrence; OS, PFS	AUC: 0.85 (training), 0.83 (validation)	Radiomic model predicts OLNM; high-risk group (via model) had worse RRFS, PFS, OS in SBRT cohort

**AI**, Artificial Intelligence; **ALC**, Absolute Lymphocytes Count; **ANN**, Artificial Neural Network; **AUC**, Area Under the Curve; **CBCT1**, the first CBCT during SBRT; **CBCTlast**, last CBCT during SBRT**; CBCTmid,** intermediate CBCT during SBRT;** CI**, Confidence Interval;** CNN**, Convolutional Neural Network;** CT**, Computed-Tomography;** DICOM**, Digital Imaging and Communications in Medicine; **Dmean**, Mean Dose;** DVH**, Dose-Volume Histogram;** GTV**, Gross Tumor Volume;** LC**, Local Control;** LN**, Lymph Node;** NSCLC**, Non-Small Cell Lung Cancer;** OLNM**, Occult Lymph Node Metastases;** OS**, Overall Survival;** PFS**, Progression-free Survival;** PTV**, Planning Target Volume;** RIIS**, Radiation-induced Immune Suppression;** RP**, Radiation Pneumonitis;** RRFS**, Regional Recurrence-free Survival;** RT**, Radiotherapy;** RV**, Right Ventricle;** SBRT**, Stereotactic Body Radiotherapy.

When considering the risk of radiation pneumonia (RP) as a consequence of SBRT for lung neoplasms, many AI-based models have been tested. Feng et al. [56] developed a model integrating radiomic features extracted from pre-treatment CT scans, dose-volume histogram (DVH) parameters and dosiomic features extracted from the 3D dose distribution for the assessment of radiation pneumonia in 140 patients with early NSCLC treated with SABR. During model development, features were progressively eliminated due to high correlation, leaving 11, 3, 25 and 9 features in the CT, CT + DVH, CT + Rtdose and hybrid models, respectively. Feature importance analysis showed that shape-related radiomic features were dominant in the CT and CT + DVH models, while GLRLM features and dosiomic parameters were among the most influential in the CT + Rtdose and hybrid models. In the results, it turned out that this new hybrid model could predict symptomatic RP better than other models based on CT-radiomics only or on CT-radiomics and DVH, with an accuracy of 0.857, a sensitivity of 1, a specificity of 0.875 and an AUC of 0.92. Kapoor et al. [57] retrospectively analyzed the data of 193 patients and developed a 3D convolutional neural networks (CNNs)-based model with input from radiographic and dosimetric datasets of primary lung tumors and surrounding lung volumes to predict the likelihood of RP. The 3D-DenseNet model reported the best performance with a F1 score of 0.81 and an AUC of 0.91 for 3-class prediction (No RP, RP1, RP 2) and an F1 score of 0.77 and an AUC of 0.84 for 2-class prediction (no RP, yes RP). To interpret the models, the authors applied Integrated Gradients (IG) heat maps, which highlighted tumor regions and their interface with lung parenchyma or pleura as critical for RP prediction. The analysis also showed contributions from surrounding lung regions, sometimes including voxels outside the tumor or dose areas, indicating that the CNN leveraged image textures and dose patterns beyond the gross tumor volume. While the heat maps provide a qualitative understanding of feature importance, the relevance of non-tumor regions to RP cannot yet be fully verified based on current physiological knowledge. Qin and colleagues [58] interestingly developed a radiomic model based on cone-beam CT (CBCT) scans both for lung injury and for PFS prediction. A total of 34 stage I NSCLC patients receiving SBRT were included in the study, and pretreatment planning CT and serial CBCT (the first during SBRT, an intermediate CBCT during SBRT and the last during SBRT) radiomic features were analyzed using the imaging biomarker explorer (IBEX) software platform. For progression prediction, key planning CT features were identified. Specifically, at baseline (planning CT), GLCM3 Variance and Global Media reflected variability in intensity and the overall mean signal. In the first CBCT (CBCT1), predictive features included measures of heterogeneity and shape, such as GLCM3 Cluster Shade, Local Entropy Max and Orientation. In mid- and last-CBCT (CBCTmid and CBCTlast), an additional textural feature (GLCM3 Information Measure Corr2) was associated with prediction. For RP prediction, planning CT features such as Shape: Mass and Shape: Orientation were significant predictors. These parameters capture both the tumor’s overall volume and the way it is positioned in space. In addition, several CBCT-derived features provided further predictive value, including NGTDM25: Contrast (reflecting local intensity differences), Shape: Max3D Diameter (measuring the largest extent of the tumor in three dimensions), ID: 60th Percentile (describing voxel intensity distribution), GLCM3: Cluster Prominence (a marker of asymmetry in texture patterns) and Shape: Convex Hull Volume (representing the geometric tightness of the tumor boundary). The model based on planning CT radiomic features achieved an AUC of 0.913 for PFS and 0.832 for toxicity. When radiomic features from CBCTs were combined with planning CT, the resulting model yielded an AUC of 0.885 for both PFS and toxicity. Notably, incorporating CBCT features alongside pretreatment CT features improved the prediction of lung toxicity compared with models relying solely on pretreatment CT. Interestingly, this was the only paper to systematically investigate CBCT-based radiomics for predicting lung injury and progression.

Bousabarah et al. [59] developed a model to predict lung fibrosis using planning CT images from 110 patients with inoperable stage I/IIa NSCLC treated with SBRT. Predictive models were successfully generated using clinical/dosimetric parameters, radiomic features, or their combination, and demonstrated consistent performance in both the training and test sets. The best-performing model incorporated four clinical/dosimetric variables (GTV-Dmean, PTV-D95%, Lung-D1ml, age) along with seven radiomic features, achieving a concordance index of 0.66 (*p* < 0.03). Among radiomic features, two stood out as most influential: Large Area Emphasis from wavelet-filtered GLSZM, which reflects the presence of large, homogeneous regions within the tumor image and the GLCM Maximal Correlation Coefficient, a texture feature that quantifies the complexity and strength of relationships between neighboring voxel intensities. Unfortunately, radiomic models failed in generalizing predictive models for oncologic outcome.

There are several limitations and potential sources of bias that should be noted in the above-mentioned studies: many papers included relatively few patients, which limits statistical power and the generalizability of the models. For example, Qin et al. [58] analyzed only 34 patients and Bousabarah et al. [59] included 110 patients. Kapoor et al. and Feng et al. had larger cohorts (193 and 140 patients, respectively), but these numbers are still moderate for AI model training and validation. Some studies did not report long-term follow-up, particularly for PFS [58] or late lung fibrosis [59]. This may underestimate late toxicity or disease recurrence and affect model evaluation. Also, most studies used internal cross-validation or test sets from the same institution. The lack of multi-center or external validation limits confidence that the models will be generalized to other populations. Patient heterogeneity (e.g., tumor location, lung function, prior treatments) and differences in imaging protocols or SBRT planning could also have influenced the results but may not have been fully accounted for.

In summary, while the studies provide promising proof-of-concept results, the relatively small sample sizes, limited follow-up, lack of external validation and potential confounding factors should be considered when interpreting the results.

When focusing on the development of cardiac toxicity following SBRT for treatment of lung cancer, Chan et al. [60] used an artificial neural network (ANN) to analyze 74 dosimetric features from 112 patients. The ANN achieved a test accuracy of 64.7% in predicting OS. Notably, patients receiving a right ventricle (RV) V10Gy dose greater than 4% had significantly shorter OS (2.4 years) compared to those receiving less than 4% (5.3 years). These findings highlight the importance of minimizing radiation exposure to cardiac substructures to improve survival outcomes in SABR-treated NSCLC patients. Nonetheless, the study is limited by small sample size, lack of distinction between cardiac and all-cause mortality and unaccounted confounders such as tumor location, histology and smoking history. Prior studies showed conflicting results: Stam et al. [64] linked higher cardiac dose to non-cancer death, while Reshko et al. [65] and Tembhekar et al. [66] found no association, the latter analyzing whole-heart dose rather than substructures. Thus, Chan et al.’s work is hypothesis-generating and notable as the first to implicate the RV specifically, but it requires validation in larger cohorts.

As SBRT can be linked to lymphodepletion, Colen et al. [61] presented an algorithm to predict radiation-induced immunosuppression (RIIS) based on a model of circulating blood using early-stage lung cancer patients treated with SBRT. The algorithm utilized treatment plan characteristics, DICOM data, dose maps and patient-specific CT datasets to model the impact of radiation on circulating lymphocytes. The model could predict post-treatment absolute lymphocyte count (ALC) with an average error of 0.24 ± 0.21 × 10^9^ cells/L. A total of 89% of patients had a prediction error below 0.5 × 10^9^ cells/L, with accurate predictions across diverse clinical and treatment factors. The model could also predict grade 2 lymphopenia (ALC < 0.8 × 10^9^ cells/L) with 81% sensitivity and 98% specificity. Overall, the algorithm showed promise for integrating with treatment planning systems to reduce immune toxicity while maintaining dosimetric quality in SBRT treatments.

With regard to outcome prediction, the work by Kim [62] validated a CT-based deep learning prognostication model in 135 patients with primary lung cancer (both squamous cell carcinoma and adenocarcinoma) undergoing SBRT. The input features to the model were only the 3D CT image patches of the primary lung tumor. Tumors were manually delineated with bounding boxes. Cubic patches (50 × 50 × 50 isotropic voxels) were extracted and intensity-normalized (HU range −1200 to 300 scaled to 0–1). No handcrafted radiomics or clinical features (e.g., stage, histology, SUVmax) were included in the input during training. Thus, the predictive features came purely from deep convolutional feature representations learned directly from tumor imaging (texture, heterogeneity, margins, internal density patterns, etc.). In multivariable Cox regression analysis, the deep learning prognostication model output remained significantly associated with survival outcomes (DFS, OS, LRFS) even after adjusting for known clinical prognostic factors (e.g., clinical T stage, nodule type, histology, BED). Deep learning model AUCs for local recurrence-free survival (LRFS), disease-free survival (DFS) and OS were 0.72, 0.70 and 0.66, respectively. The model therefore provided useful independent information beyond clinical factors.

Ni et al. [63] also focused on the development and validation of a CT-based radiomic model to predict occult lymph node metastasis (OLNM) and assess the risk of regional recurrence (RR) in patients with clinical stage I NSCLC treated with SBRT. Using data from 769 surgically treated patients, the radiomic model demonstrated strong predictive performance (AUC 0.85 in the training set, 0.83 in the validation set) without improvement from adding clinical features. When applied to 213 SBRT-treated patients, those classified as high-risk by the model had significantly higher RR, shorter regional RFS, PFS and OS. These results suggest that radiomic-based risk stratification may guide personalized surveillance and treatment strategies in early-stage NSCLC.

### 3.2. Integration Between AI and SBRT for Treatment Assessment and Outcomes Prediction in Hepatobiliary Cancer

**Figure 3:**
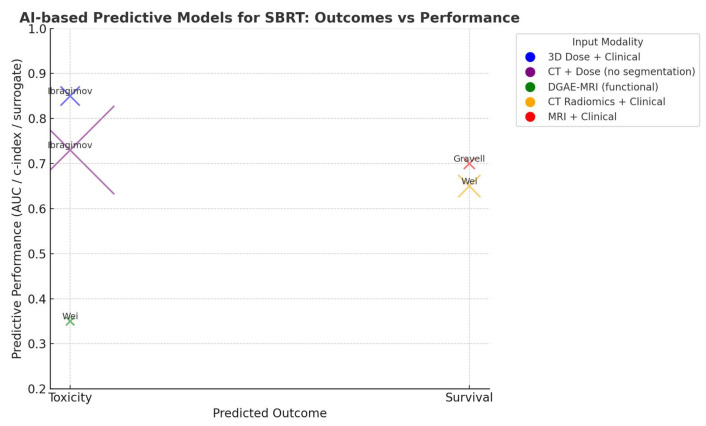
Bubble chart summarizing the main findings of studies evaluating AI models in liver cancer patients treated with SBRT. The *x-axis* represents the predicted clinical endpoints (treatment-related toxicities and oncological outcomes). The *y-axis* indicates the predictive performance reported (AUC or accuracy). Bubble size is proportional to the number of patients included in each study, and bubble colors distinguish the different input modalities used. Labels correspond to the first author of each study. This visualization provides an at-a-glance overview of the heterogeneity of AI approaches, their sample sizes and performance across toxicity and outcome prediction tasks.

Five selected papers from 2018 to 2024 dealt with AI-based models for outcomes prediction and toxicity assessment in patients treated with SBRT for pancreatic or hepatic neoplasms (Table 2, Figure 3).

**Table 2:** Artificial Intelligence-based models for outcomes assessment and toxicity prediction in patients undergoing SBRT for hepatobiliary cancer.

First Author	Year	Imaging Modality	Type of Used AI	N. Patients	Outcomes Assessed	Predictive Performance (AUC, Accuracy, Sensitivity)	Main Findings
Ibragimov B. [67]	2018	CT	Deep Learning	125	Toxicity	CNN hepatobiliary toxicity prediction: AUC 0.79. Combined CNN for 3D dose plan analysis and fully CNN for numerical feature analysis: AUC 0.85. Irradiation of the proximal portal vein associated with two times higher toxicity risks (risk score: 0.66) than irradiation of the left portal vein (risk score: 0.31)	Clinically accurate tools for HB toxicity prediction and automatic identification of anatomical regions critical to spare during SBRT were provided
Ibragimov B. [68]	2020	CT	Deep Learning (CNN)	122	Toxicity	CNN model toxicity prediction accuracy: AUC 0.73. Significantly higher risk scores (*p* < 0.05) of HB toxicity manifestation associated with irradiation for the hepatobiliary tract in comparison to the risk scores for liver segments I–VIII and portal vein	Without any prior anatomical knowledge, CNNs automatically recognized the importance of hepatobiliary tract sparing during liver SBRT
Wei L. [69]	2023	MRI	Machine Learning Deep Learning	24	Toxicity	D50 was about 35.2 Gy for the general group, but patients with worse liver function had a D50 of only 11.7 Gy. Patients with better liver function had a higher D50 of 54.8 Gy. The machine learning model was able to predict how the liver would respond during treatment and its predictions matched well with the real results	This study successfully developed models that use MRI scans to predict how much liver function will be lost during radiation treatment for HCC patients
Wei L. [70]	2021	CT	Deep Learning	167	OS	The combined model (using both radiomics and clinical data) reported a c-index of 0.650. The most important features for predicting survival were liver function and the radiomic features of the liver outside the tumor	The study found that combining radiomics, clinical factors and deep learning models provides better predictions of overall survival for HCC patients than traditional methods
Gravel R. [71]	2024	MRI	Machine-Learning	41	EFS	Cox model: Harrell’s C-index—training: 0.78, test: 0.94	ML model using MRI and clinical data (age, albumin, intra-lesional fat) predicts EFS after SABR in HCC

**AI**, Artificial Intelligence;** AUC**, Area Under the Curve;** CCA**, Cholangiocarcinoma;** CNN**, Convolutional Neural Network;** EFS**, Event-free Survival;** HB**, Hepatobiliary;** HCC**, Hepatocellular Carcinoma;** ML**, Machine Learning;** MRI**, Magnetic Resonance Imaging;** OS**, Overall Survival;** SABR**, Stereotactic Ablative Radiotherapy;** SBRT**, Stereotactic Body Radiotherapy.

Three works investigated toxicity predictive models; amongst them, Ibragimov et al. [67] collected a database of 125 liver SBRT cases with follow-up data to train a deep learning-based model for predicting toxicity. Convolutional neural networks (CNNs) were employed to identify consistent patterns in 3D dose plans linked to toxicities with CNN first pretrained using transfer learning on 3D CT images of 2644 human organs to improve prediction accuracy before being trained specifically on liver SBRT cases. To improve prediction, the authors incorporated also non-dosimetric pretreatment factors—such as patient demographics, underlying liver disease and prior liver-directed therapies—into a fully connected neural network. Using only the CNN to analyze 3D dose distributions yielded an AUC of 0.79 for predicting hepatobiliary toxicity. However, when the CNN outputs were combined with the additional clinical features in a joint model, the prediction accuracy increased to an AUC of 0.85. In 2020, the same author [68] investigated whether CNNs could predict post-radiotherapy toxicities by automatically linking abdominal CT images and RT dose plans, thus eliminating the need for manual organ segmentation. The study generated risk maps for different liver anatomical regions and statistically compared them with existing clinical data to assess their validity. The CNNs were trained to identify acute and late grade 3 or higher toxicities, including biliary stricture/obstruction, hepatic failure or decompensation, hepatobiliary infections, elevated liver function tests and portal vein thrombosis. The models achieved a toxicity prediction performance with an AUC of 0.73. Risk scores for hepatobiliary toxicity were significantly higher (*p* < 0.05) when the hepatobiliary tract was irradiated, compared to scores for liver segments I–VIII or the portal vein. Another model developed by Wei et al. [69] exploited pre- and during-RT dynamic Gadoxetic Acid-enhanced (DGAE)-MRI in a cohort of 24 patients; liver function was quantified using the contrast uptake rate (k1) from MRI and a logistic dose–response model estimated liver function loss based on changes in liver reserve (Child-Pugh score). In the results, the dose causing 50% loss of liver function (D50) was 35.2 Gy, but for patients with poor baseline liver function, D50 was significantly lower at 11.7 Gy, indicating higher radiosensitivity.

Two works on the other hand investigated predictive models related to survival outcomes; Wei et al. [70] conducted a retrospective analysis of 167 patients and extracted 56 radiomic features from pre-treatment contrast-enhanced CT scans and 37 clinical factors from patient records. A model combining both radiomic and clinical data yielded a c-index of 0.650, with liver function and radiomic features from the liver (excluding the tumor) being the most important factors. Deep radiomic analysis could improve HCC prognosis prediction compared to traditional Cox models and may aid in patient stratification and treatment personalization, with liver function being a key contributor to OS. In line with these results, Gravell and colleagues [71] explored the feasibility of using a machine learning-based predictive model to estimate event-free survival (EFS) in patients with HCC treated with SABR. A total of 41 patients and 64 lesions were analyzed, with patients split into training and test cohorts. Three ML models were developed and the best-performing one—a Cox regression model—used pre-treatment variables such as age, albumin levels and intra-lesional fat identified on MRI. Therefore, combining clinical and MRI-derived features with ML may provide accurate outcome predictions in HCC patients undergoing SABR, supporting more personalized treatment planning.

Overall, these studies demonstrate that AI-driven models integrating imaging biomarkers, clinical data and dosimetric variables can significantly improve the prediction of both toxicity and survival outcomes in hepatobiliary cancer patients undergoing SBRT.

The conventional, non-AI-based guidelines for SBRT to the liver are primarily based on dose–volume constraints derived from large multi-institutional datasets and expert consensus. For example, QUANTEC recommends limiting the mean liver dose to <15–18 Gy in patients with adequate baseline function (Child-Pugh A) and ensuring that at least 700 cc of uninvolved liver receives <15 Gy [72], in order to minimize the risk of classic radiation-induced liver disease (RILD). These guidelines provide population-level safety thresholds, but they do not fully account for individual variability in baseline liver function, comorbidities or regional differences in radiosensitivity. By contrast, the AI-based models we reviewed aim to move beyond this one-size-fits-all approach, which suggests a potential role for personalized adaptation beyond QUANTEC-style constraints.

In summary, while current non-AI guidelines remain essential for safe SBRT planning, AI-driven models offer complementary, patient-specific risk stratification that could help to individualize dose prescriptions, particularly in patients with compromised liver function.

### 3.3. Integration Between AI and SBRT for Treatment Assessment and Outcomes Prediction in Brain Cancer

**Figure 4:**
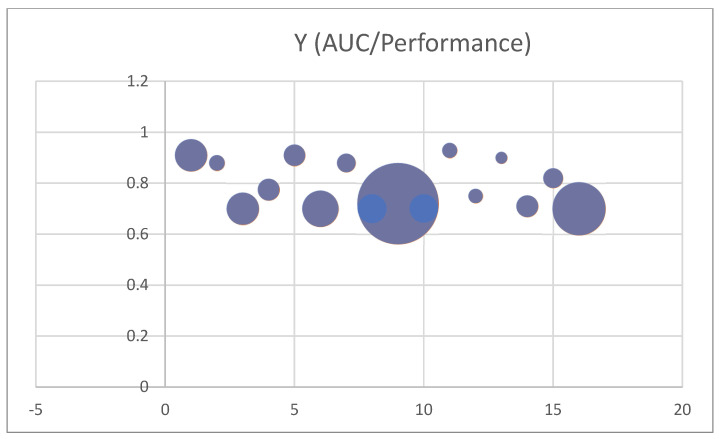
Bubble chart summarizing the main findings of studies evaluating AI models in CNS neoplasms treated with SBRT. The *X-axis* shows outcomes numbered from 1 to 16 according to the table’s order, while the *Y*-axis indicates predictive performance (AUC or accuracy). Bubble size corresponds to the number of patients included in each study. Outcomes are represented numerically for visualization purposes. For studies where AUC or accuracy was not reported, a default value of 0.7 was used. This visualization provides an at-a-glance overview of the heterogeneity of AI approaches, their sample sizes and performance across toxicity and outcome prediction tasks.

Sixteen papers investigated AI-based models for outcomes prediction and post-treatment toxicities in different heterogeneous brain tumors, also including benign neoplasms (Table 3, Figure 4).

**Table 3:** Artificial Intelligence-based models for outcomes assessment and toxicity prediction in patients undergoing SBRT for heterogeneous brain tumors.

First Author	Year	Imaging Modality	Type of Used AI	N. Patients	Outcomes Assessed	Predictive Performance (AUC, Accuracy, Sensitivity)	Main Findings
Huang PW [73]	2022	MRI	Unsupervised Machine Learning	209	Obliteration and RIC	Compact AVM (OR 3.12, 95% CI 1.01–9.61) was a positive predictor, whereas AVM volume (OR 0.96, 95% CI 0.94–0.98) and deep venous drainage (OR 0.38, 95% CI 0.17–0.85) were negative predictors of complete obliteration after GKRS. Compact AVM (OR 0.33, 95% CI 0.13–0.82) was an independent negative predictor of RIC	The compactness index quantitatively described the compactness of unruptured AVMs. Compact AVMs may have a higher obliteration rate and a smaller risk of RICs than diffuse AVMs
Huang PW [74]	2024	MRI	Classical statistical modeling	262	Post-SRS hemorrhage	Post-SRS hemorrhage rate increased with larger AVM volume only among the diffuse nidi (1.7 vs. 14.9 vs. 30.6 hemorrhage per 1000 person-years in AVM volume <20 cm^3^ vs. 20–40 cm^3^ vs. >40 cm^3^; *p* = 0.022)	Compact and smaller AVMs, with higher prescribed margin dose, harbored lower risks of post-SRS hemorrhage. The post-SRS hemorrhage rate exceeded 2.2% annually within the diffuse and large (>40 cm^3^) AVMs and the diffuse Spetzler-Martin IV–V AVMs
Gao D. [75]	2022	MRI	Machine Learning	88	Oncological Outcome	Radiomic model (12 features) AUC: 0.88 (95% CI 0.87–0.90). Two radiomic features, “Dependence Variance” and “First-order Skewness”, were significant between early or late responders	Radiomic features can be used for the pretreatment prediction of outcome for GKRS in unruptured AVMs
Huang CY [76]	2023	MRI	Machine Learning	330	Pseudo-progression	Likelihood of pseudo-progression after GKRS for solid vs. cystic VS: 55% vs. 31%, *p* < 0.001. For the entire cohort, MVA revealed that a lower mean tumor SI in T2W/CET1W images was associated with pseudo-progression after GKRS (*p* = 0.001)	Pseudo-progression is more likely to occur in solid vs. compared with cystic vs. quantitative radiological features in pretreatment MRI were associated with pseudo-progression after GKRS
Langenhuizen PPJH [77]	2020	MRI	Machine Learning	99	TTE	Patient- and treatment-related characteristics were not correlated with TTE. First-order statistical features and Minkowski functionals from the MRI also did not help predict TTE. However, a set of 4 GLCM features reported 82% sensitivity and 69% specificity, with even better performance for tumors greater than 6 cm^3^ (sensitivity 77% and specificity 89%)	MRI tumor texture can provide valuable information for predicting TTE
George-Jones N. [78]	2021	MRI	Machine Learning	53	Tumor Enlargement >20%	The tumor shape and texture features-based model had a sensitivity of 92%, specificity of 65%, AUC of 0.75 and a positive likelihood ratio of 2.6 (95% CI 1.4–5.0)	VS shape and texture features may be useful inputs for machine learning models that predict vs. enlargement after SRS
Lee S. [79]	2016	MRI	Machine Learning	702	Communicating HCP	Significant risk factors for developing communicating HCP were older age (*p* = 0.0011); vestibular origin (*p* = 0.0438); larger tumors (*p* < 0.0001). The ML model showed higher risk of communicating HCP with vestibular schwannomas and tumors larger than 13.65 cm^3^	HCP is not a rare complication after GKRS for intracranial schwannomas, especially in older patients, those with vestibular-origin tumors and those with larger tumors
Moon HC [80]	2024	MRI	Machine Learning	80 (training set), 40 (test set)	3-month OS	Decision tree accuracy: 77.5% Random forest accuracy: 72.5% Boosted Tree classifier accuracy: 70% The most important factors for survival predictions were age and chemotherapy, significant across all algorithms. Tumor volume (larger than 10 cc) was another key factor	The decision tree algorithm was the most accurate and showed that patients older than 71 years and with a tumor volume larger than 10 cc were at higher risk of dying within 3 months after GKRS
Jalalifar SA [81]	2023	MRI	Deep Learning	96 (training set), 20 (test set)	LC/LF and ARE	Deep learning-based longitudinal segmentation demonstrated a good agreement with manual assessment with an accuracy, sensitivity and specificity of 91%, 89% and 92%, respectively, for LC/LF and 91%, 100% and 89% in detecting ARE on the independent test set	Implementation of the proposed system in clinical settings can potentially accelerate longitudinal tumor size analyses and streamline image-guided therapy outcome evaluation workflows
Keek SA [82]	2022	MRI	Supervised Machine Learning	1404 (training cohort), 237 (test cohort)	ARE	Different XGBoost models were developed using only radiomics features, only DL features, only patient characteristics or a combination of these features. At lesion-level, the best-performing model combined radiomics and DL features, with an AUC of 0.71. At patient level, the highest performance was achieved by combining radiomics features, DL features and patient characteristics, resulting in an AUC of 0.72	Machine learning models integrating radiomics, DL features and patient data show promise in predicting ARE risk in patients undergoing GKRS for BM
Jaberipour M. [83]	2020	CT	Machine Learning	120	Local failure	The AdaBoost classifier with decision tree reported a sensitivity, specificity and accuracy of 76.9%, 66.7% and 71.0%, respectively, for prediction of LC/LF	Noncontrast quantitative CT with machine learning can predict LC/LF outcome in metastatic brain tumors treated with SRT at pre-treatment
Sharma M [84]	2025	MRI	Classical statistical modeling	262	RN risk after repeated SRS	NTCP models: AUC up to 0.91 (clustered feature method with SVM)	Recurrent brain metastases have lower dose tolerance threshold and more gradual dose response; modeling time-discounted cumulative dose improves prediction accuracy
Zhao J. [85]	2025	MRI+ genomic and clinical data	Deep Learning	62	Differentiation of RN vs. tumor recurrence post-SRS	AUC 0.88 ± 0.04; Sensitivity 0.79 ± 0.02; Specificity 0.86 ± 0.01; Accuracy 0.84 ± 0.01	Heavy Ball Neural ODE (HBNODE) model integrating multimodal data outperformed image-only and other combined models; this model also provided explainability by tracking feature importance over time
Qiao N. [86]	2022	NA	Deep Learning	58	Time to Endocrine Remission	A machine learning model combining pathology images with clinical and genetic data reported 92.9% accuracy rate in the test dataset	By combining pathology images with clinical and genetic information, the AI model was much better at predicting endocrine outcomes for acromegaly patients after radiosurgery than traditional methods
Kim KH [87]	2022	MRI	Deep Learning	202	PTE	Hybrid data model accuracy and AUC: 0.725 and 0.701, respectively. The performance of the hybrid data model was superior to that of the other models based on clinical or image data only	DNN-based model using both clinical and imaging data exhibited fair results in predicting post-GKS PTE in meningioma treatment
Goyal S. [88]	2021	NA	Deep Learning	36	Efficacy	ANN model: 90% accuracy on 11 tested cases. A greater number of Pre-GKT medications, previous MVD, V2 dermatome involvement and negative history of post-GKT numbness were negative prognostic factors	Lesser pre-GKRS drugs used, involvement of V1 dermatome, post-GKT numbness are favorable prognostic factors. Failed MVD for TN is associated with poor outcome, as well as repeated GKRS

**AI**, Artificial Intelligence;** ANN**, Artificial Neural Network;** ARE**, Adverse Radiation Effect;** AVM**, Artero-Venous Malformation;** AUC**, Area Under the Curve;** BM**, Brain Metastases;** CET1W**, Contrast-enhanced T1-weighted;** CI**, Confidence Interval;** CT**, Computed Tomography;** DL**, Deep Learning;** DNN**, Deep Neural Network;** GKRS**, Gamma Knife Radiosurgery;** GKT**, Gamma Knife Treatment;** GLCM**, Grey-Level Co-occurrence Matrix;** HCP**, Hydrocephalus;** LC**, Local Control;** LF**, Local Failure;** ML**, Machine Learning;** MRI**, Magnetic Resonance Imaging;** MVA**, Multivariate Analysis;** MVD**, Microvascular Decompression;** NTCP**, Normal Tissue Complication Probability;** OS**, Overall Survival;** PTE**, Peritumor Edema;** RIC**, Radiation-induced Changes;** RN**, Radionecrosis;** SRS**, Stereotactic Radiosurgery;** SRT**, Stereotactic Radiotherapy;** SI**, Signal Intensity;** SVM**, Support Vector Machine;** T2W**, T2-weighted;** TN**, Trigeminal Neuralgia;** TTE**, Transient Tumor Enlargement; VS, Vestibular Schwannoma.

For what concerns arterio-venous malformations (AVMs), three works were included in the present analysis; the first one, by Huang et al. [73], evaluated AVM compactness and assessed its impact on survival outcomes following GKRS. The researchers indeed differentiated tissue components (vessel, brain, cerebrospinal fluid) on T2-weighted MRI scans and introduced a compactness index as the ratio of vascular to brain tissue. Based on this index, AVMs were categorized into compact, intermediate and diffuse types. Compact AVMs, smaller AVM volume and superficial venous drainage were independent predictors of higher obliteration rates; on the other hand, diffuse AVMs, larger volume and higher margin dose were linked to an increased risk of radiation-induced changes (RIC). Consistently with these findings, Huang et al. [74] conducted a retrospective study to examine whether the morphology of AVMs—classified as compact or diffuse—was associated with the risk of hemorrhagic events in patients undergoing SRS for unruptured AVMs. Among diffuse AVMs, the rate of post-SRS hemorrhage increased with AVM volume (1.7, 14.9 and 30.6 hemorrhages per 1000 person-years for volumes < 20 cm^3^, 20–40 cm^3^ and >40 cm^3^, respectively; *p* = 0.022). In contrast, smaller, compact AVMs treated with higher prescribed margin doses exhibited a lower likelihood of post-SRS hemorrhage. Finally, the paper provided by Gao and colleagues [75] explored a radiomic model from 12 selected features from pre-treatment MRI scans and its correlation with obliteration and RIC. The model showed strong performance with an AUC of 0.88, outperforming traditional scoring systems. Four higher-order texture features reflecting heterogeneity/homogeneity of the AVM nidus (GLSZM_Zone Entropy (ZE); GLSZM_Short Zone Non-Uniformity Normalized (SZNN); GLSZM_Gray Level Non-Uniformity (GLN); and GLDM_Dependence Non-Uniformity Normalized (DNN)) reported lower values in the favorable outcome group (obliteration), suggesting more homogeneous lesions had better response. Additionally, two radiomics features, “Dependence Variance”, which captures spatial heterogeneity, and “First-order Skewness,” which captures overall asymmetry in voxel intensity distribution, were found to significantly differ between early and late responders, based on obliteration within two years.

Four works dealt with patients affected by vestibular schwannoma (VS). In the most recent, published by Huang and colleagues [76], data from pre-treatment T2W/contrast-enhanced T1-weighted image (CET1W) of 330 patients treated with GKRS for VS were correlated with pseudo-progression development. Radiological features were derived from T2W/CET1W images after fuzzy C-means clustering-based classification of VS into solid and cystic types. For the entire cohort and solid VS subgroup, the main feature was the mean tumor signal intensity (SI) in T2W/CET1W images. For the cystic VS subgroup, additional features included the mean SI of the solid and cystic components, the proportion of the cystic component (cystic volume/total tumor volume) and shape features of the cystic component (sphericity, flatness, elongation). Pseudo-progression was more likely to occur in solid VS compared with cystic VS, thus highlighting the potential of quantitative radiological features of pre-treatment MRI in predicting treatment response after GKRS. Transient tumor enlargement (TTE), a possible adverse effect following GKRS for VS, was assessed by Langenhuizen et al. [77]. Indeed, the authors analyzed clinical data of 99 patients and extracted first-order statistics, Minkowski functionals (MFs) and three-dimensional GLCMs from the treatment scans. While first-order statistical features (mean, standard deviation, skewness, kurtosis and histogram-based metrics) and Minkowski functionals (threshold-dependent morphological descriptors) from the MRI did not help to predict TTE, a set of 4 GLCM features (entropy, contrast, energy, correlation) reported 82% sensitivity and 69% specificity, with even better performance for tumors greater than 6 cm^3^ (sensitivity 77% and specificity 89%). Therefore, MRI tumor texture could be useful in TTE prediction and in establishing the most tailored treatment strategy for each patient. In a similar study, George-Jones et al. [78] analyzed texture and shape characteristics from SRS planning scans of 53 patients and employed these features to train a linear support vector machine classifier to predict post-SRS volume increase exceeding 20% of the pretreatment size. The developed model reported a sensitivity of 92%, a specificity of 65%, an AUC of 0.75 and a positive likelihood ratio of 2.6 (95% CI 1.4–5.0) for predicting post-SRS enlargement of >20%. Tumor shape and size features were the most predictive of enlargement in smaller VS, while texture features were more predictive in larger tumors. Back in 2016, Lee and colleagues [79] aimed to identify the incidence and the risk factors for communicating hydrocephalus (HCP) after GKRS for intracranial schwannomas. In their retrospective review of 702 patients treated between 2002 and 2015, they reported that 29 patients (4.1%) developed communicating HCP, requiring ventriculo-peritoneal (VP) shunt surgery. At multivariate analyses, significant predictors for HCP turned out to be older age (*p* = 0.0011), tumor origin (*p* = 0.0438) and larger tumor volume (*p* < 0.0001). Specifically, VSs with a volume ≥ 13.65 cm^3^ had the highest risk.

Brain metastases were assessed in six papers: Moon and colleagues [80] investigated a machine learning algorithm for 3-month OS assessment in a cohort of patients undergoing GKRS for brain metastases from NSCLC. The most important factors for survival predictions resulted age and chemotherapy use; also, tumor volume (larger than 10 cc) was another key factor. The study by Jalalifar [81] presented as well a deep learning-based system for automatic assessment of outcomes in brain metastasis treated with SBRT using MRI. Trained on 96 patients, it accurately detected tumor control/failure (91%) and adverse radiation effects (ARE) (91%) in an independent test set, showing strong agreement with expert evaluations. The involved features were primarily tumor size measurements derived from automatic segmentation masks. Specifically, these included the longest diameter of the tumor in the axial, coronal and sagittal planes at baseline and follow-ups; the relative change in longest diameter compared to baseline and nadir; the tumor volume at each time point and relative change in volume compared to baseline and nadir.

These features were then used to automatically determine tumor size status (increase, stable, decrease), local control or failure and ARE outcomes, following clinical criteria such as RANO-BM and volumetric response guidelines. The prediction of AREs was central in the paper by Keek [82], where ML models were exploited to assess radiation-induced toxicity in brain metastasis patients even before the administration of RT. Using gadolinium-enhanced MRI data and patient characteristics, the study trained models based on radiomics, deep learning and their combination. In the end, the best model combined both radiomics and DL features, providing an AUC of 0.72 and recall of 0.84 at the patient level. Radiomic shape features (especially LeastAxisLength), certain first-order and texture features, deep learning features and patient characteristics such as age and prior treatment were consistently among the most predictive. When considering local control in the setting of brain metastases treated with SBRT, Jaberipour [83] investigated if the adoption of quantitative CT biomarkers and machine learning could predict local failure. The best model, using AdaBoost with decision trees, predicted local failure with 71% accuracy on an independent test set. Local failure prediction was associated with Shape 3D Elongation, a feature that quantifies how elongated or stretched the tumor shape is in three dimensions, and GLDM 3D Dependence Non-uniformity Normalized, which reflects the variability in voxel groupings of similar intensity values, thereby capturing the degree of heterogeneity within the tumor texture.

Sharma et al. [84] developed and compared normal tissue complication probability (NTCP) models for recurrent brain metastases treated with second SRS, incorporating the effect of time-dependent discounting of prior radiation dose. Using Gamma Knife data, the models revealed that recurrent tumors have a lower dose tolerance and a more gradual dose response to retreatment compared to non-recurrent lesions. Importantly, accounting for the diminishing effect of the first treatment over time improved the accuracy of predicting radio-necrosis (RN) risk. These findings highlight the potential of advanced NTCP modeling to tailor SRS retreatment protocols as well. When dealing with brain metastases and the risk of RN, Zhao et al. [85] developed a deep learning model, Heavy Ball Neural Ordinary Differential Equation (HBNODE), to accurately differentiate RN from tumor recurrence in NSCLC brain metastases treated with SRS. By integrating longitudinal MRI data with clinical and genomic information from 62 patients (90 lesions), the model achieved a high predictive performance (AUC 0.88) and outperformed models based on imaging or clinical data only. Patient age, use of chemotherapy/targeted therapy and use of immunotherapy predominantly influenced the model’s predictions in the early stages, whereas imaging-derived features (from DNN image inputs) became dominant later in the trajectory, indicating that spatial or radiomic characteristics of the lesions were more predictive as the model processed intermediate states. Overall, genomic features never dominated the prediction; they had only a supplementary role compared to clinical and imaging features.

Pituitary adenomas, meningiomas and trigeminal neuralgias (TN) were underrepresented in our series.

Qiao et al. [86] interestingly applied AI to analyze pathological images (*pathomics*) for predicting outcomes in acromegaly patients after SRS and obtained a model which could predict endocrine remission with 92.9% accuracy, 87.5% sensitivity and 100% specificity, with proliferation (Ki-67), cellularity, granulation pattern and some immunohistochemistry markers (p53+, SSTR2A-) being the main pathomic features. For what concerns meningiomas, Kim et al. [87] developed a deep neural network model to assess peritumoral edema (PTE) after GKRS for meningioma using data from 202 patients, including clinical and imaging information. Post-GKS PTE occurred in 13.9%, and the hybrid model combining clinical and imaging data achieved an accuracy of 72.5% and an AUC of 0.701, outperforming those algorithms using only clinical or imaging data. Finally, Goyal et al. [88] evaluated the long-term outcomes, prognostic indicators and complications of GKRS in trigeminal neuralgia and developed an artificial neural network model. They identified several negative prognostic factors, including a higher number of medications prior to GKRS, a history of microvascular decompression, involvement of the V2 dermatome and the absence of post-GKRS numbness.

AI-based models, integrating radiomic, clinical and functional imaging data, show strong potential in brain tumors treated with SRS. They may enhance risk stratification, toxicity prediction and response assessment across both malignant and benign intracranial conditions. Particularly in AVMs, schwannomas and brain metastases, AI might enable more tailored treatment strategies, potentially improving outcomes and minimizing adverse effects.

After highlighting the characteristics of the included studies, Table 4 reports the assessment of risk of bias, evaluated with the Newcastle–Ottawa Scale (NOS). Overall, most of the studies included in the analysis demonstrated a low risk of bias, reinforcing the reliability of the findings. Nonetheless, a considerable number of studies showed a moderate risk, which warrants caution in the interpretation of the results.

**Table 4:** Quality evaluation of included studies using the Newcastle Ottawa Scale (NOS) for cohort studies.

Author, Year	Selection	Comparability	Outcome	NOS Score
Colen J, 2024 [61]	***	*	***	7
Feng A, 2024 [56]	****	**	***	9
Ni J, 2024 [63]	****	**	***	9
Kapoor R, 2017 [57]	****	**	***	9
Bousabarah K, 2021 [59]	****	**	***	9
Kim H, 2021 [62]	****	**	***	9
Chan ST, 2020 [60]	****	*	***	8
Qin Q, 2020 [58]	***	*	***	7
Gravel R, 2024 [71]	***	*	***	7
Wei L, 2023 [69]	***	*	**	6
Wei L, 2021 [70]	***	**	**	7
Ibragimov B, 2020 [68]	***		**	5
Ibragimov B, 2018 [67]	***		**	5
Sharma M, 2025 [84]	***	*	**	6
Zhao J, 2025 [85]	***	*	**	6
Huang PW, 2024 [74]	***	**	**	7
Moon HC, 2024 [80]	***	*	**	6
Jalalifar SA, 2023 [81]	***	*	**	6
Huang CY, 2023 [76]	***	*	**	6
Gao D, 2022 [75]	***	*	***	7
Huang PW, 2022 [72]	***	*	***	7
Keek SA, 2022 [82]	***	*	***	7
Kim KH, 2022 [87]	***	*	***	7
Qiao N, 2022 [86]	***	**	***	8
George-Jones N, 2021 [78]	***	**	***	8
Goyal S, 2021 [88]	***	*	***	7
Jaberipour M, 2020 [83]	***	*	***	7
Langenhuizen PPJH, 2020 [77]	***	*	***	7
Lee S, 2016 [79]	***	*	***	7

In a NOS scale the asterisks represent the score that can be attributed to each item under evaluation.

## 4. Discussion

The results of our review confirmed that AI-based predictive models show promising results for their integration into clinical practice for outcomes and toxicities assessment in different heterogeneous solid tumors, primary or metastatic, intracranial or extracranial, treated with either SRS or SBRT.

The integration of AI into Oncology practice is rapidly evolving, not only as a diagnostic support tool but also as a means to tailor patient management in real-world applications. Recent studies, such as that by Gunisetty et al. [89], demonstrate the potential of AI-based apps in optimizing chemotherapy and lifestyle management, suggesting a broader role for AI in personalized cancer care, including SBRT/SRS workflows.

AI may also play a role in the optimization of treatment technique; in this regard, Rawal et al. [90] demonstrated the dosimetric advantages of VMAT over IMRT in head and neck cancers, underscoring the potential for AI-driven radiomics to further optimize such techniques in SBRT/SRS.

In the setting of lung malignancies, characterization through radiomics and deep learning is becoming increasingly crucial to detect nodules, differentiate between malignant and benign lesions and assess lesion histology, stage and genotype [20,40].

Deep learning models as well have contributed to improve automated segmentation of organs at risk in lung cancer radiotherapy [91,92], to allow stratification of patients according to their risk of local and distant recurrence and to determine the most suitable candidates for molecular-targeted treatments [44] and immunotherapy. SBRT is currently considered a standard of care option for patients with early-stage lung cancer who are medically inoperable or refuse to undergo surgery. Results from our review outline the potential of AI-driven models to improve the prediction of outcomes and toxicities in lung cancer patients undergoing SBRT. Many studies focused on the development of radiation pneumonitis, while others explored survival outcomes. CT-based radiomic models were most adopted, often empowered by the incorporation of other dosimetric, clinical or additional imaging data. Hybrid approaches have showed superior performance, with higher accuracy and AUC values.

Several recent non-systematic literature reviews have previously reported the impact of AI in the context of lung SBRT. In the work by Avanzo et al. [93], radiomic models have demonstrated the ability to assist in the early identification of potential toxicities. In this context, changes in CT radiomic features from pre- to post-treatment (at 3, 6 and 9 months), influenced by both dose and fractionation schedule, were found to significantly correlate with radiation-induced lung injury (RILI) following SBRT. Moreover, radiomics has shown potential in the early prediction of tumor recurrence, which typically manifests after one year, based on CT scans obtained approximately 3 and 6 months post-SBRT. As early as 2015, Avanzo et al. already explored threshold and optimal dose levels for differentiating tumor recurrence from RILI during follow-up. Their findings indicated that dosiomics—the integration of dose features derived from the dose distribution in the irradiated lung calculated from the planning CT—was predictive of RILI [94].

In a previous systematic review, Walls et al. [95] explored the utility of Radiomics in predicting local control, survival outcomes and toxicity after SBRT for lung cancer. Their analysis indicated that radiomic features were more effective in predicting local failure than regional or distant failure and that combining PET and CT features improved predictions of local control in SABR cohorts. For pulmonary toxicity, they found that radiomic features from pre-treatment breath-hold planning CT scans could help identify patients at risk of developing post-SABR local fibrosis. Additionally, features within the same dataset were associated with local failure, disease-free survival and overall survival. All the aforementioned evidence was built on CT-based imaging, while in a recently published narrative review [96], Cheng and colleagues exploited the role of MRI-guided SBRT for the treatment of lung cancer. This technique has recently growing potential thanks to its inherently superior soft tissue visualization capability [97], which can be leveraged to minimize cardiac irradiation exposure and to enhance the precise delineation of ultra-central primary tumors and metastatic lymph nodes. In this context, the implementation of a novel deep learning-based super-resolution technique in T1-weighted volume-interpolated breath-hold examination (VIBE) post-contrast imaging has led to improved image quality, reduced noise levels and increased diagnostic confidence, with shortened acquisition time [98]. In their work, the authors showed how the introduction of MRI-guided SBRT for lung cancer offers daily adaptive capability and real-time target tracking, as well as the ability to assess response to treatment early by exploiting quantitative MRI (qMRI).

Regarding hepatobiliary neoplasms, specifically liver tumors, the advent of advanced radiation techniques such as SBRT and IGRT has enabled radiotherapy to effectively manage both primary and metastatic lesions, achieving encouraging local control rates. By incorporating multiparametric quantitative data, including advanced radiomic analyses, Radiation Oncology can now access information that goes beyond the qualitative assessment of visible changes [99].

From our results, combining CT-radiomics, clinical factors and deep learning models provided better predictions of overall survival for HCC patients than traditional methods.

Much of the literature evidence is available regarding the role of Radiomics, both AI-and non AI-based, in predicting local response after Trans-Arterial Chemo-Embolization (TACE) [100] and after Volumetric Modulated Arc Therapy (VMAT) [101] for HCC and liver metastases. By combining radiomics features with clinical data, survival prediction might be improved in patients with HCC [43,102,103] and, even in case of cholangiocarcinoma, pre-operative MRI features were able to predict early recurrence, along with immunohistochemical markers [104].

When dealing, on the other hand, with liver toxicity following SBRT, a dose constraint of sparing at least 700 cm^3^ from receiving over 15 Gy across three fractions has shown low toxicity rates, validated in various Phase I and II trials [6,105,106,107]. Predicting liver toxicity following SBRT involves analyzing both dosimetric and non-dosimetric factors due to the complex interactions influencing toxicity risk (pre-existing comorbidities, concurrent treatments, liver function), and machine learning is therefore increasingly seen as a promising approach to model these intricate relationships.

From our results, both MRI- and CT-based radiomic features proved useful for prediction of remaining liver function after SBRT and of hepatobiliary toxicities (e.g., biliary stenosis). As already shown by Toesca et al. [108], successful toxicity prediction depends on identifying relevant features linked to toxicity and on choosing algorithms—like random forests or neural networks—that can uncover consistent patterns. Dosimetric features often highly correlate with toxicity, as seen in other models for radiation-induced pneumonitis and gastrointestinal toxicities. Unfortunately, machine learning models specific to RILD prediction are limited and future studies should integrate liver dosimetry, pre-existing liver conditions (e.g., cirrhosis, HCV, HBV), radiogenomic data and concurrent therapies to enhance RILD prediction.

Regarding pancreatic cancer, not mentioned in our review, there is a growing body of literature regarding the role of CT-based radiomic models for grade prediction of neuroendocrine neoplasms [42], for the assessment of liver metastases [109], for predicting early distant relapses after upfront surgery [110] and for radiological–pathological correlation studies [111].

In the setting of brain tumors, most of the available literature concerns the application of ML methods in the management of brain metastases. During the diagnostic process, MRI algorithms have already proven useful in the distinction between high-grade glioma and solitary brain metastases [112,113]. In addition, in our series, the application of AI-based models has demonstrated a potential role in overall survival and local control prediction and in the assessment of AREs.

In line with our findings, Habibi et al. [114] performed a systematic review and meta-analysis of 17 studies, seven of which focused on using ML to predict local failure and eleven on predicting treatment response in brain metastases treated with SRS. The ML models showed a sensitivity of 0.89 (95% CI: 0.84–0.93) and a specificity of 0.87 (95% CI: 0.81–0.92) for treatment response prediction. For local failure, the pooled sensitivity and specificity were 0.93 (95% CI: 0.76–0.98) and 0.80 (95% CI: 0.53–0.94), respectively. The authors concluded that ML shows significant potential for predicting treatment outcomes and local failure in patients with brain metastases undergoing SRS, though further research and refinement are needed before these models can be fully integrated into clinical practice.

Again, according to the literature search by Kocher et al. [115], “classic” Radiomics represented a promising field for predicting response in brain metastases after SRS, with simple features (presence of a necrotic core, fraction of contrast-enhancing tumor tissue, extension of the perifocal edema) showing an impact on response or survival. In the systematic search by Kanakarajan [116], factors associated with better local control in brain metastases treated with SRS and resulting from a machine learning application were breast cancer primary type, uterine cervical carcinoma histology, absolute neutrophil count, serum albumin concentration, lymphocyte percentage, higher radiation dose, higher volume coverage, addition of Whole-Brain Radiotherapy (WBRT), single-fraction, Tyrosine-kinase inhibitors, up-front SRS, higher number of radiation shots, previous craniotomy and Renal Cell Carcinoma (RCC)-specific Graded Prognostic Assessment (GPA) score. For fractionated SRT, factors associated with better LC turned out to be higher radiation dose, prior surgery, adenocarcinoma as histological type, tumor volume decrease after the first SRT and higher KPS.

For what concerns AVMs, our series reported the results from three different retrospective works, mostly focusing on vascular compactness and on the dimension of nidi to predict prognosis and toxicities following SRS. Currently, there is no available literature evidence regarding the use of prognostic and predictive AI-based models in the context of AVMs treated with radiosurgery. The adoption of AI in this setting was reported only by Colombo et al. [117] who systematically reviewed current approaches for 3D segmentation and visualization of AVMs based on 33 studies. The authors categorized segmentation methods as automatic, semiautomatic or manual, noting that automatic algorithms were the most used (61%) and generally required less time. Our series reported four studies involving the role of AI in response prediction (including the risk of pseudo-progression) and in toxicity assessment following SRS when dealing with vestibular schwannomas, a setting where AI has been exploited in the past years for recurrence prediction [118], for patients’ stratification [119], for nerve function prediction following surgery [120,121,122].

Our review comprehensively evaluates AI models also for the management of intracranial meningiomas, pituitary adenomas and trigeminal neuralgia. This represents a novel contribution to the field, as no prior reviews have systematically explored the application of AI-based predictive models in these peculiar clinical settings. Again, our work underscores the potential for AI to enhance decision-making and personalize care across a wide range of pathologies treated with SRS.

When evaluating the contribution of AI in SRS/SBRT outcome prediction, it is important to distinguish between studies that mainly confirm already well-recognized clinical dependencies and those that introduce novel imaging or biological biomarkers. For lung cancer, many studies confirmed already recognized clinical factors such as tumor size, heterogeneity and dose metrics. For example, Bousabarah et al. [59] and Kim et al. [62] showed that target dose, lung exposure and tumor imaging heterogeneity remain strong predictors of control and toxicity, while Kapoor et al. [57] essentially automated established dose–volume–outcome relationships using CNNs. By contrast, other works introduced less conventional biomarkers that extend beyond size or simple DVH analysis: dosiomics captured spatial dose heterogeneity and improved RP prediction over DVH alone; on-treatment CBCT radiomics provided dynamic biomarkers predictive of progression and toxicity; cardiac substructure dose, particularly RV V10Gy, associated with survival after thoracic SBRT; mechanistic blood-dose modeling predicted radiation-induced immune suppression with high specificity and CT radiomics for occult nodal disease identified a high-risk group with inferior survival outcomes. These studies highlight how AI can enrich prediction by adding new feature classes (dosiomics, substructure dosimetry), new time points (serial CBCT) or new biological constructs (circulating lymphocyte dose).

In liver neoplasms as well, several models reflect already established dependencies, such as Wei et al.’s [69] ML model confirming that baseline liver function strongly determines tolerance to radiation, though quantified more precisely (D50 ≈ 11.7 Gy in poor vs. 54.8 Gy in preserved liver function). At the same time, Gravel et al. [71] and Ibragimov et al. [67] uncovered novel imaging or AI-derived biomarkers, such as MRI parenchymal features and automatically identified hepatobiliary substructures, expanding prediction beyond traditional clinical factors.

Similarly, in brain tumors, many findings aligned with known dependencies: Moon et al. [80] linked larger tumor volume and older age with poor OS, while Lee et al. [79] confirmed tumor size and vestibular origin as predictors of communicating HCP. Yet, other works introduced new quantitative imaging features, including radiomic descriptors (“Dependence Variance” and “First-order Skewness”) for AVM response [75], GLCM texture features for time-to-enlargement [77] and compactness index as a predictor of obliteration and radiation-induced changes [73]. These studies illustrate AI’s potential not only to reaffirm and more precisely quantify already known predictors, but also to reveal novel radiomic and structural biomarkers.

Concrete examples of the clinical impact of AI models include optimizing radiation doses to minimize toxicity and supporting patient selection for SBRT versus alternative therapies. In early-stage lung cancer, AI-driven radiomic and dosiomic models can predict the risk of radiation pneumonitis, allowing clinicians to adjust dose distributions to spare healthy lung tissue. Similarly, in liver tumors, predictive models can estimate residual liver function and help to prevent radiation-induced liver disease by guiding dose constraints. AI can also support treatment planning by identifying the most suitable irradiation technique, such as VMAT versus IMRT, based on tumor characteristics and OARs considerations. For brain metastases, ML algorithms can predict treatment response and local recurrence risk and guide the choice between SRS, fractionated regimens or combined systemic therapies.

It should be outlined that most of the AI models reported in the present review are not publicly available. The majority of models are indeed developed and validated internally at single institutions, often using proprietary clinical and imaging datasets. This reflects a common challenge in AI applications in SBRT and SRS, due to patient privacy, institutional data ownership and regulatory constraints

The integration of AI into clinical practice is hindered also by the variability in medical imaging data which arises from differences in imaging protocols, scanner types and patient demographics across institutions. Such inconsistencies can negatively affect the performance and generalizability of AI models. To address these challenges, two pivotal strategies have been proposed: the establishment of standardized imaging protocols and the implementation of federated learning frameworks. The former is crucial for ensuring consistency in medical imaging data. Recent studies have emphasized the importance of harmonizing imaging protocols to face these issues. For instance, a systematic review [123] highlighted strategies for image harmonization, which ensures consistency and improves reliability of pooled data analysis, thus enabling AI analysis of multi-source medical imaging. Additionally, current overviews [124] have been proposed to facilitate standardization in preprocessing and harmonization, addressing limitations in Radiomics and enhancing the robustness of AI models. Federated learning (FL), on the other hand, offers a promising solution to the data privacy concerns inherent in medical imaging. This decentralized approach allows multiple institutions to collaboratively train AI models without sharing sensitive patient data. A comprehensive survey [125] on federated learning methods for medical image analysis underscores its potential in preserving privacy while enabling collaborative learning across heterogeneous datasets. Moreover, real-world implementations have demonstrated the efficacy of FL in improving model generalizability. For example, a study on breast density classification showed that models trained using FL performed better and exhibited improved generalizability across different institutions compared to those trained on local datasets alone [126].

In conclusion, the clinical translation of AI in medical imaging can be significantly enhanced by adopting standardized imaging protocols and federated learning frameworks. These approaches not only address the challenges posed by data variability and privacy concerns but also pave the way for the development of robust, generalizable AI models that can be seamlessly integrated into clinical practice.

Furthermore, some aspects of our review continue to raise concerns. First of all, the relatively small number of included studies—all retrospective—and limited patient sample sizes may impact the robustness of our findings. Finally, anticipated advancements in radio-genomics are crucial to deepen our understanding of underlying biological processes, including intrinsic radiosensitivity [127]. Radio-genomics is indeed an emerging field that integrates imaging features from radiological studies, such as CT, MRI or PET scans, with genomic and molecular characteristics of tumors. By correlating image-based traits with genetic or molecular profiles, radio-genomics may enable non-invasive insights into tumor biology, support the prediction of treatment response, guide personalized therapy and help to identify patients at higher risk of toxicity or recurrence. This approach represents a promising avenue for enhancing precision medicine in oncology.

Despite these limitations, the existing evidence is promising and justifies further exploration, as the demonstrated predictive accuracy for common toxicities shows considerable potential benefits.

## 5. Conclusions

The integration of AI into SBRT might represent a paradigm shift toward precision medicine. While further validation is needed, the convergence of imaging, clinical and biological data through AI holds the promise to improve outcomes, reduce toxicities and guide individualized treatment strategies.

## Supplementary Materials

The following supporting information can be downloaded at: https://www.mdpi.com/article/10.3390/cancers17172906/s1, Figure S1: PRISMA check-list.

## Abbreviations

The following abbreviations are used in this manuscript:

AI	Artificial Intelligence
ALC	Absolute Lymphocytes Count
ANN	Artificial Neural Network
ARE	Adverse Radiation Effect
AUC	Area Under the Curve
AVM	Arterio-venous Malformation
BED	Biological Equivalent Dose
CBCT	Cone-beam Computed Tomography
CCA	Cholangiocarcinoma
CET1W	Contrast-enhanced T1-weighted
CI	Confidence Interval
CNN	Convolutional Neural Network
CT	Computed Tomography
D_50_	Dose that causes a 50% effect
D_mean_	Mean Dose
DGAE	Dynamic Gadoxetic Acid-enhanced
DICOM	Digital Imaging and Communications in Medicine
DL	Deep Learning
DNN	Deep Neural Network
DFS	Disease-free Survival
DVH	Dose-Volume Histogram
EBRT	External Beam Radiotherapy
EFS	Event-free Survival
FL	Federal Learning
GKRS	Gamma Knife Radiosurgery
GLCM	Gray-Level Co-occurrence Matrix
GLN	Gray Level Non-Uniformity
GLRLM	Gray-Level Run Length Matrix
GPA	Graded Prognostic Assessment
GTV	Gross Tumor Volume
GY	Gray
HB	Hepatobiliary
HBNODE	Heavy Ball Neural Ordinary Differential Equation
HCC	Hepatocellular Carcinoma
HCP	Communicating Hydrocephalus
IBEX	Imaging Biomarker Explorer
IG	Integrated Gradients
IGRT	Image-guided Radiotherapy
IMRT	Intensity-modulated Radiotherapy
KPS	Karnofsky Performance Status
LRFS	Local Recurrence-free Survival
ML	Machine Learning
MRI	Magnetic Resonance Imaging
NGLDM	Neighborhood Gray-Level Difference Matrix
NOS	Newcastle Ottawa Scale
NSCLC	Non-Small Cell Lung Cancer
NTCP	Normal Tissue Complication Probability
OAR	Organs at Risk
OLNM	Occult Lymph Node Metastasis
OS	Overall Survival
PET	Positron Emission Tomography
PFS	Progression-free Survival
PRISMA	Preferred Reported Items for Systematic reviews and Meta-Analyses
PTE	Peritumoral Edema
PTV	Planning Target Volume
QUANTEC	Quantitative Analysis of Normal Tissue Effects in the Clinic
RIC	Radiation-induced Changes
RIIS	Radiation-induced Immunosuppression
RILD	Radiation-induced Liver Disease
RILI	Radiation-induced Lung Injury
RN	Radiation Necrosis
ROC	Receiver Operating Characteristics
ROI	Region of Interest
RP	Radiation Pneumonitis
RR	Regional Recurrence
RT	Radiotherapy
RV	Right Ventricle
SABR	Stereotactic Ablative Radiotherapy
SBRT	Stereotactic Body Radiotherapy
SRS	Stereotactic Radiosurgery
SRT	Stereotactic Radiotherapy
SUV	Standard Uptake Volume
SZNN	Short Zone Non-Uniformity Normalized
T2W	T2-weighted
TACE	Trans-Arterial Chemo-Embolization
TN	Trigeminal Neuralgia
TTE	Tumor Transient Enlargement
VIBE	Volume-interpolated Breath-hold Examination
VMAT	Volumetric Modulated Arc Therapy
VOI	Volume of Interest
VP	Ventriculo-peritoneal
VS	Vestibular Schwannoma
WBRT	Whole-Brain Radiotherapy
ZE	Zone Entropy
